# Exploring perceptions, attitudes and beliefs of Thai patients with type 2 diabetes mellitus as they relate to medication adherence at an out-patient primary care clinic in Chiang Mai, Thailand

**DOI:** 10.1186/s12875-020-01233-7

**Published:** 2020-08-21

**Authors:** Wichuda Jiraporncharoen, Kanokporn Pinyopornpanish, Korsin Junjom, Nisachol Dejkriengkraikul, Anawat Wisetborisut, Iliatha Papachristou, Ahmar Hashmi, Chaisiri Angkurawaranon

**Affiliations:** 1 Department of Family Medicine, Faculty of Medicine, Chiang Mai University, 110 Intawaroros Road, Sriphum, Muang, Chiang Mai, 50200 Thailand; 2 Department of Epidemiology and Population Health, London School of Hygiene and Tropical Medicine, Keppel Street, London, WC1E 7HT UK

**Keywords:** Type 2 diabetes mellitus, Medication adherence, Self-management, Health behavior, Family practice

## Abstract

**Background:**

Within the sphere of diabetes self-management, much emphasis has been placed on medication adherence. There has been a shift in thinking about medication adherence, moving from “compliance” and historically paternalistic models of care, to seeking better ways of characterizing dynamic and complex relationships that determine medication adherence and diabetes control. This study sought to understand the relationship between patient’s attitudes and medication adherence for oral anti-diabetics in Thailand.

**Methods:**

In-depth interviews of patients with type 2 diabetes mellitus, taking oral anti-diabetic drugs, at the out-patient clinic run by the Department of Family Medicine, Chiang Mai University between May and December 2016. Thematic analysis followed the WHO framework for medication adherence in chronic disease to explore patient’s attitudes and their influence on medication compliance.

**Results:**

Of 24 patients, 9 were men. The mean age was 62 years (SD 8.9 years). 67% had high compliance. Four themes were identified as important factors related to medication adherence: attitudes toward disease, attitudes toward treatment, attitudes toward family support and attitudes toward health care team. Specifically, symptoms at diagnosis, understanding and acceptance in taking medication, the presence of family support and the perception of concern by the doctor relate to improved medication compliance.

**Conclusions:**

Medication adherence in Thai patients with diabetes requires support from both the health care providers and the family. The patient’s perception of the doctor’s concern creates greater patient trust in the health care team. This trust, along with family support, helps deepen patients’ understanding of the disease, accept the chronic nature of their disease, and engenders a positive attitude towards taking medication that can improve medication adherence.

## Background

Type 2 diabetes mellitus (T2DM) affects populations worldwide. In 2017, there were an estimated 425 million (CI 346–545 million) people between 20 and 79 years globally with diabetes and it is predicted to rise to nearly 630 million affected and a prevalence nearing 10% by 2045 [1].

This has become a formidable health challenge facing low- and middle-income countries (LMIC) such as Thailand. Trebling over the previous two decades, there were an estimated 4 million Thai adults living with diabetes by 2015 [2, 3]. Diabetes is the cause of death in 11,389 per 100,000 deaths, has one of the highest prevalence in the Western Pacific Region nearing 8% among 20–79 year-olds, and is the 5th leading cause of disability in Thailand [3, 4]. Studies have demonstrated the considerable social and economic costs associated with T2DM in Thailand [5, 6].

Given Thailand’s strong primary health system and universal health coverage, medical management of T2DM is widely available [2]. However, surveys of patients with diabetes in Thailand document that less than half achieve optimal control of clinical risk factors for disease: with 33% of patients achieving optimal HbA1C levels; 44% maintaining appropriate LDL cholesterol levels; and 52% with appropriate blood pressure control. Regular follow-up and screening for microvascular complications is similarly limited, with fewer than 60% of patients with diabetes in Thailand receiving comprehensive annual screening [2, 7]. However, T2DM management and preventing its complications requires not only access to and utilization of facility-based services, but also appropriate self-management of disease. T2DM patients therefore need to incorporate a number of lifestyle changes that allow for medication adherence, appropriate diet, and adequate exercise. Given the chronic nature of this disease, patients must develop the knowledge, skills and abilities for adequate self-management [8].

Within this sphere of T2DM self-management, much emphasis has been placed on medication adherence. There has been a shift in thinking about medication adherence, diverging from notions of “compliance” and historically paternalistic models of care, to seeking better ways of characterizing dynamic and complex relationships that determine medication adherence and diabetes control. The WHO, in highlighting the need for a stronger health systems approach to medication adherence for chronic disease, asserted that medication adherence in diabetes hinged upon five key factors: therapy-related factors, condition-related factors, health systems/health care team (HCT)-related factors, patient-related factors, and social/economic-related factors [9]. Therapy-, condition-, and health systems/HCT-related factors consider the complexity of the medication regimen, the duration of the disease, delivery of care, and patient-provider relationships. In addition to age and gender, other patient-related factors such as self-esteem, self-efficacy, depression and alcohol abuse can impact medication adherence. Social/economic-related factors often consider cost of medicines and care, as well as social support networks.

Thailand, as in other LMIC, has few robust studies characterizing lifestyle factors as they impact anti-diabetic medication adherence and T2DM management. Studies conducted in Thailand have attempted to make the link between T2DM indicators and patient adherence to medication, but these studies do not provide a convincing, plausible relationship due to limitations in their design [10, 11].

Our study aims to further explore the relationship between attitudes and medication adherence in patients with diabetes taking oral anti-diabetics. Overall, we hope to enhance the explanatory power of perceptions of Thai patients with diabetes and their medication adherence. We also aim to identify the facilitators and barriers of patients with low adherence compared to those patients with high adherence.

## Methods

### Study design, location and participants

A qualitative, case study exploratory approach was used for this study. In-depth interviews of patients with type 2 diabetes was conducted by using semi-structured guides at the Outpatient Department of the Department of Family Medicine, Chiang Mai University, at Maharaj Nakorn Chiang Mai Hospital, Chiang Mai, Thailand. Participants were selected based on the following inclusion criteria: (a) patient age at least 20 years old, with a diagnosis of type 2 diabetes (confirmed by ICD-10 in the hospital record system); (b) taking oral anti-diabetic drugs, and (c) proficient in Thai language. Convenience sampling was used, aiming for at least 20 participants at which point saturation was expected based on previous studies [12–14].

### Data collection

Data collection consisted of 2 parts: (1) participant characteristics and (2) in-depth interviews exploring perceptions and attitudes toward diabetes as they relate to participant symptoms, medications, diagnosis, health care and physician or health care staff interactions. Each interview took between 30 and 45 min. Interviews were conducted in Thai by a female study nurse trained in qualitative interviewing with 3 years experience in conducting qualitative interviews. The interviewer was not directly involved in the usual care at the clinic. Members of the research team observed the interviews (WJ and/or KJ) and participants consented to having the interviews recorded.

Participant characteristics included age (< or ≥ 60 years) and gender. Level of adherence to medication was assessed by asking 3 questions: “Do you take medication regularly?” “How often do you forget?” and “Have you ever stopped taking medication by yourself?” Adherence was categorized into two groups, “low” or “high”, with high adherence defined as taking medication regularly, missing a dose ≤ 3 times in a month, and not prematurely discontinuing medications without physician approval.

In-depth interview guides were created from medication adherence for chronic disease as outlined by WHO [9], and focused on the five factors associated with adherence to anti-diabetic medications (Table 1). It was adapted to the local context by the research team (K.J., K.P. and W.J.). These guides, written in Thai language, were piloted with a small number of patients, then corrected and finalized.

**Table 1:** In-depth interview guide and the specific aspects of anti-diabetic medication adherence covered

Factor discussed	Questions
**1. Condition-related factors**	
• The symptom at DM diagnosis	How were you diagnosed with T2DM
	What were your symptoms?
• Perceived causes of T2DM	What do you think caused your T2DM?
• Feelings related to a T2DM diagnosis	When you were diagnosed with T2DM, were you concerned? Why or why not?
**2. Therapy-related factors**	
• Perceptions of long-term medication treatment	What do you think or feel about having to take medication long-term for anti-diabetic treatment?
• Perception of the amount and frequency of medication used	How many pills do you take daily?
	Do you think this is appropriate or too much? Why or why not?
**3. Health system/Health care team (HCT)-related factor**	How is your relationship with your doctor or healthcare team?
**4. Social/economic-related factors**	Who do you stay with?
	Do they know about your T2DM diagnosis?
	How does your family support you?
**5. Patient-related factors**	How often do you forget to take your medicine? why?
	Have you ever decide to stop by yourself and why?

### Data analysis

Descriptive statistics were used for participant characteristic. Qualitative analysis followed the WHO framework, which provided a priori codes and definitions, with emergent codes developed by W.J. and K.P. following review of transcripts from the first few IDI. K.J. then performed line-by-line inductive analysis according to the agreed upon codes immediately after each subsequent IDI. W.J. reviewed and corroborated analysis for K.J. using a small set of transcripts, and updated the codebook accordingly. This iterative process helped identify when and if saturation had been reached, at which point data collection was stopped and no further participants interviewed. Transcripts were then reviewed by K.J. according to the finalized codebook, who performed preliminary textual analysis with particular attention paid to level of adherence (low vs high), gender, and/or age. W.J. and K.J. reviewed and performed thematic analysis, which was then reviewed, discussed, and finalized with other investigators (A.W., K.P., N.D., A.H., I.P, C.A.).

## Results

### Participant’s characteristics

At total of 24 participants were interviewed between May and December 2016. Participants had a mean age of 62 years, with 38% ≥ 60 years of age, and 62% female. Sixty-six percent (16/24) were classified as having high adherence (Table 2).

**Table 2:** Participant characteristics, (*n* = 24)

Factor	
Age Mean age (SD)	62 (8.9)
< 60 years (n, %)	15 (62.5%)
≥ 60 years (n, %)	9 (37.5%)
Sex (n, %)	
Male	9 (38%)
Female	15 (62%)
Adherence (n, %)	
Low	8 (33.3%)
High	16 (66.7%)

**Table 3:** Themes and subthemes of IDI analysis following the WHO framework for medication adherence

Themes	Sub-themes
(1) Attitudes toward disease	**Symptoms at diagnosis and concerns**
	• Neurological symptoms: headache, dizziness, stroke
	• General symptoms: fatigue, thirsty, frequent urinate
	• Asymptomatic with incidental DM diagnosis
	**Effect to ADLs/Non effect to ADLs**
	**Health belief related to disease causation**
	- Irreversible condition (risk form older, genetic disease)
	- Behavior (lack of exercise, poor diet control, obesity)
	- Cause from hypertension and dyslipidemia
	- Unknown
(2) Attitudes toward treatment	**Daily medication prescription**
	- Afraid of adverse effects from medications, especially renal side effects
	- Understand and accept medical treatment
	**Adverse effect associated with treatment**
	- Has experienced
	- Has experienced but corrected by health care team
	**Number of medications taken perceived as:**
	- Too many
	- Appropriate
(3) Attitudes toward family support	- No family involvement or reminders to take medication
	- Family support in various ways
(4) Attitudes toward health care team	**Providing treatment and advice**
	- Fear of diabetic complications
	- Trust doctor’s advice
	**Doctor-patient relationship**
	- Doctor demonstrated compassion and understanding

### Factors related to medication adherence

Originally conceived as five factors affecting adherence, patient- and condition-related factors were combined upon analysis given that participants often discussed these factors together. Thus, four themes were identified: attitudes toward disease (patient- and condition-related factors), attitudes toward treatment (therapy-related factors), attitudes toward family support (social- and economic-related factors) and attitudes toward health care team (health systems/HCT-related factors) (Table 3). These themes were compared for participants with low and high adherence. Additional quotes supporting the thematic analysis can be found in supplementary Table 1.

#### Attitudes toward disease

Participants’ attitudes toward disease were impacted by symptoms (or lack thereof) experienced at the time of diagnosis, understanding of disease causation, pathophysiology, or natural progression of disease. Importantly, more severe disease, greater personal concern about and/or negative experiences with disease symptoms or sequelae prompted better patient adherence to anti-diabetic medications.

*“I felt fatigue and my head was dizzy and felt tense all day. I had tried herbs for 2 days but they didn't work well. I didn’t know what was happening to me. I felt really sick. I never had these symptoms before, so I went to the doctor. At that time, I thought that I needed to be admitted to the hospital. After the doctor checked and found that my blood sugar was 400 [mg/dl] and my blood pressure was around 200 [mm Hg]...I have continued the medication regularly. I am afraid to forget taking it because I don't want my blood sugar to increase again. Since being diagnosed with diabetes, I have good control of my diet. I don't eat snacks, sweets. I changed from sticky rice to normal rice. I have increased vegetables and decreased fried food****.”***

***—***female, high adherence

As expected, symptoms (or lack thereof) could help determine if patients took medications. The following quote is particularly illustrative: a participant reports not having diabetes symptoms at the time of his diagnosis, is lulled by the lack of symptoms into poor medication adherence, subsequently suffers a terrible symptomatic event (stroke), which impresses upon him the importance of taking anti-diabetic medications.

"*I was diagnosed with diabetes about 7 years ago after a regular checkup. I was not surprised as I was quite fat at that time. I wasn’t very concerned because I thought my health was very good and I never had any abnormal symptoms. So I decided not to take any medication or see the doctors for 2 years. Then it was suddenly worse when I suffered a stroke. At that time, I thought it was a really terrible condition. I could not live without help from other people, even get dressed or button my shirt. That caused me great concern…I think diabetes is just as bad as cancer.*"

-- male, high adherence.

#### Attitudes toward treatment

Attitudes toward treatment focused predominantly on taking medications, including the number of medications required, their effects on disease sequelae, and associated side effects. Concerns about long-term side effects of medications—such as the risk of renal failure—made participants reluctant to take medication. High adherers believing the medication necessary and/or able to prevent diabetic complications were more accepting of the frequency and dosing of anti-diabetic medications.

*"I know I have to take anti-diabetic medication forever. This disease cannot be cured. I will take it regularly until I die. I meet the doctor at every appointment and take the pills when I return home. If I have to visit my daughter in Japan, I will ask for more pills. I never forget to take it. I take 6 pills for diabetes and 1 for dyslipidemia."*

*Facilitator: "Do you think is it too many?"*

*"The amount of medication is lower than before....It's necessary to take them. Some people who don't have other diseases might take fewer pills than me. It's reasonable because I have underlying disease. I want to live a long with my family so I can take care of them."—* female, high adherence

However, low adherers had multiple reasons for not taking medications, chief among them being: medication adherence as burdensome; belief in alternative or traditional cures; fear of medication side effects; and fatalistic perspectives with regard to their diagnosis.

*"I take anti-diabetics medication, 4 tablets, and also one for hypertension and another one for hyperlipidemia for a total of 6 tablets a day. I usually leave anti-diabetics medication for the evening. I don't want to take it. I think it is too much. If I didn't have other diseases, I won't have any problems with taking it. I also take herbs and hope that if my blood sugar gets better I can stop taking these anti-diabetic drugs. I fear that my kidneys will be destroyed. I saw people get hemodialysis from renal failure. I fear to be like that. The doctor told me to take medication regularly, but if I forget just sometime at that evening--it will not be a big problem, right?"*

*—*female, low adherence

This quote exemplifies the kind of nuanced feelings espoused by many participants with low adherence. Comorbidities require a larger number of medications that need to be taken over the course of a day, which can be quite burdensome. Here we find that not only are alternative therapies sought, but that they arise from the desire for a cure hatched from a potential misconception regarding the participant’s diagnosis. This contrasts with the high-adhering participant above, who internalizes the lack of cure as central to her need to take medications regularly. Another consistent theme—among both low and high adherers alike—was the fear of medication side effects, especially their effects on the kidneys. Importantly, complications such as diabetic nephropathy—the most common cause of renal failure—didn’t seem to stoke as heightened a fear for kidney health compared to fears of medication-induced nephropathy. These misunderstandings are described in detail by another participant with low adherence:

*"Right now, I take 2 tablets for diabetes, 1/2 tablet for dyslipidemia and 1/2 tablet for hypertension. I also take several herbs. My family suggests that these herbs could lower blood sugar. My sister was diagnosed with diabetes and used to take anti-diabetic medication and suffered many side effects. After taking herbs, these abnormalities had gone and their blood sugar was controlled. The first time I took medication, I didn’t feel good, sometimes I had palpitations and dizziness. It was too many pills, my body couldn't tolerate it, so I stopped taking them for a while. After that, the doctor adjusted the medication and I had to be admitted to the hospital several times from side effects. These days, I am fine. I am OK with the amount of my medication. I take less than before. In fact, I don't want to take anti-diabetic medications. I think it can accumulate in my body and end up with kidney disease or other consequences. I saw my uncle, he took a lot of medication around 6-7 tablets every day until he suffered renal failure and required a kidney transplant. I notice that anyone who takes medication, they are never cured from diabetes even taking it every day...like my uncle. You know…he had taken it every day. I never saw his blood sugar controlled and worse than that--he had to inject insulin every day****."***

—female, low adherence

Here we can identify how patients with diabetes may ascribe beliefs and understandings about the disease to their experienced reality. This patient in particular followed the example of family members and their family’s well-intentioned advice. However, there seems to be some confusion in discerning effects of the disease and its complications from medication side effects—or even those of the herbal supplements. Although this participant’s physician addressed potential side effects from medications, there was still considerable disbelief that anti-diabetic medicines prevent future sequelae. Finally, as this quote exemplifies, participants often hinted at a creeping fatalism about their condition that medications would be unable to stymie the eventuality of diabetic complications or, possibly, death.

#### Attitudes toward family support

There were various ways in which families supported patients that included reminding them to take medication, helping prepare medications, cooking proper meals, and taking participants to the hospital. In addition, participants noted that the love and care shown to them by family members motivated participants to take care of themselves by taking their medications.

*“When one of us [participant or the participant’s husband] has free time, we will prepare our own medication and each others, so we can know who forgets taking their pills. We usually take medication together after breakfast. He helps me a lot.”—* female, high adherence

*“My children usually remind me to not eat sweets or fatty foods and to eat more fruits and vegetables. They helped me prepare medication in the beginning but now I can do by myself. They usually remind me not to forget to take medication every day.”*— female, high adherence

Particpants suggested that not having the support of attentive family members may in fact relate to poor medication adherence:

*“I prepare medication by myself, no one helps me. My wife reminds me sometimes....once in a while...It would be nice if she asked me every day. I understand she is busy....has a lot of work to do.”*— male, low adherence.

#### Attitudes toward the health care team

The nature and character of the patient-physician relationship proved important in understanding medication adherence among participants. Having a favorable attitude toward one’s physician, participants’ perceived compassion and understanding from one’s physician, or feeling as though the doctor was an integral member of the diabetes care team, could encourage participants to take medication. Participants who believed they required a physician’s help in spite of their self-management often were concerned about the seriousness of their disease or feared that they did not have a deep understanding of diabetes. These participants sought out a physician as a reliable source of advice. With such trust came a desire to adhere to the health care teams’ recommendations, including treatment.

*"I decided to start anti-diabetic medication upon my doctor’s advice. In fact, I can still do only lifestyle modification. I know that I am quite old right now and should do as the doctors suggest mostly. They are specialized in this way, however, I wonder about my own situation of having DM, and ask, "Why do I have this disease?" and, "Is the suggestion from the doctor clear*?"— male, high adherence

*"I don't stress about taking anti-diabetic medication. The number that I should take is up to the doctor’s suggestion. It' s important to believe the doctor, so I take medication regularly. Someone may stop medication by themselves if their blood sugar is improve but not me. I want to hear from my doctor first. They are very nice to me. With these good advice and their support, I feel 100% that I can control my DM. Sometimes I work out-of-town as a tour guide, but I still make sure to take the medication that the doctor prescribed."*— male, high adherence

Although both high and low adherers perceived that the health care team provided knowledge of disease and treatment, those with good adherence more often reported a stronger feeling of empathy and understanding on the part of their physician.

## Discussion

This study sought deeper understanding into the adherence to oral anti-diabetic medications among patients with T2DM in Thailand, through a qualitative approach that utilized a known WHO framework for understanding factors that can enhance or impede medication adherence. Four main themes were related to adherence to medication regimens, including patients attitudes toward the disease itself (patient- and condition-related factors); attitudes toward medications (therapy-related factors); attitudes toward social support (social/economic-related factors); and attitudes toward the health care team (health systems/HCT-related factors). The most common impediments to taking the anti-diabetic medications related to misconceptions about the disease itself and often stemmed from distrust of anti-diabetic medications, and preference for traditional therapies in lieu of doctor-prescribed regimens. Perceived symptoms, particularly at the time of diagnosis or when experiencing diabetic complications, often related to improved medication adherence. A strong family support system could enhance medication adherence and overall awareness for Thai patients with diabetes. Compassion from health providers and feeling like an invested partner in the patient-physician relationship could further compel patients to take medications more regularly.

For this discussion, we focus on similarities between ours and other studies in factors affecting medication adherence among people with diabetes, and highlight some implications for improving medication adherence in a Thai context.

### Attitudes toward disease

In this study, attitudes toward diabetes and the need to take medications related to having severe symptoms prior to diagnosis and/or having symptoms that disturbed activities of daily living (ADLs). As in a study by Brundisini et al., Thai participants' fears of the harms of T2DM—either through immediate disruptions to ADLs or long-term sequelae—served to heighten the participants’ desire to improve medication adherence [15]. By contrast, lacking overt symptoms of disease may have the opposite effect, similar to studies elsewhere that note that “normal” blood sugar levels were interpreted by patients to mean that they did not have to take medicines [16, 17] .

With regard to symptoms (perceived or not), and other attitudes that can lead to low medication adherence, patient education may be the most promising means to dispel misconceptions and engender more positive attitudes toward self-management [18, 19]. Helping patients understand the pathophysiology of the disease can help discern side effects related to medications compared to symptoms of disease, and can assuage fears of medication-induced nephropathy. We note that participants who viewed diabetes as an incurable but manageable condition not only understood the importance of adhering to medical regimens, but also espoused healthy lifestyle choices such as limiting sugar and fat consumption. Helping patients understand this distinction may curb fatalistic views of the disease. Although not specifically emerging from our dataset, we are reminded that stigma related to diabetes can make patients reluctant to intiate treatment and maintain adherence [18].

### Attitudes toward treatment

Findings from this study echo studies highlighting poor medication adherence due to fears of side effects and a preference for herbs or local alternatives. Fears among participants that long-term treatment would cause renal failure led to intentional and/or unconsciously poor medication adherence. We found that the “burden” associated with oral anti-diabetic medications was largely perceived instead of being associated with the actual number of medications to be taken, as participants who understood medication was necessary in preventing diabetic complications could accept whatever amount of medication needed. Negative attitudes toward anti-diabetic medications cultivated a sense among participants that oral anti-diabetics were too burdensome regardless of the amount, leading to conscious or unconscious non-adherence. As in a meta-analysis of T2DM patients’ and providers’ differing perspectives on medication nonadherence, this study also finds that intentional non-adherence was an effort to avoid perceived side effects of anti-diabetic medications [15]. A study by Sohal et al. characterized barriers and facilitators for T2DM management in South Asia, noting misconceptions such as reduced or missed doses could reduce medication side effects, and a preference for herbal or traditional treatments perceived as safer than western medications [20].

As in other studies, our study implies that more conscious efforts to counter beliefs that medication side effects make people sicker can improve medication adherence [17, 19]. An important example of this is the fear of hypoglycemia, which has been attributed as a cause for patients to stop or self-adjust regimens [21], and adverse events associated with anti-diabetics have made patients in neighboring Malaysia [22] and further afield in Bangladesh [13] reluctant to adhere to therapeutic regimens. In our study, some participants experienced medication side effects but were able to continue medication treatment after their physician adjusted their medication regimen. Therefore, in addition to allaying fears of medication side effects, clear open discussions between patients and their physicians in the event of side effects is essential. Multi-faceted strategies to promote adherence may be tailored to the Thai context and focus on education to adresss misconceptions and advise on missed doses, medication reminders, and feedback on the impact of medication on glycemic control [18].

### Attitudes toward family support

Our study findings are similar to studies identifying the supportive actions families of patients with diabetes can take to enable higher quality of life and better control of the disease and its complications [23–25]. Such actions include encouraging and supportive communication, and practical considerations found in our study such as managing diet, medications, and home blood glucose monitoring [23]. Not only were these family interactions important for high adherence among our study participants, but their *very absence* seemed to risk poor adherence.

Hence, as education seems the likely implication of our findings in the Thai context, encouraging family members to get involved in diabetic treatment as part of the care team can be very helpful [26]. Families are crucial for managing chronic disease [25]—possibly all the more important in LMIC settings in Asia as demonstrated in Thailand, Cambodia, and India [5, 27, 28]—and a systematic review suggests that developing interventions to enhance family support of patients with diabetes can improve health outcomes of patients with uncontrolled glycemic levels [24].

### Attitudes toward health care team

The study highlights that the relationship a patient has with their health care team and their physician plays an important role in Thai patients adherence to medications. As in other Asian communities, we suspect that Thai patients often consider the physician as the authoritative source of diabetes knowledge and management [20]. However, this implies a power dynamic that may prevent patients from disclosing failure to comply with their physician’s recommendations. For Thai patients, stronger perceived empathy from the health care team and their physicians was noted among high adherers. This is something to be strived for as evidence from Bangladesh suggests that a lack of empathy and reassurance from participants’ physicians were barriers to effective diabetes management [29]. Training multidisciplinary care teams, including nurses, dieticians, pharmacists, and social workers in counselling and motivational interviews could be useful to enhance self-management skills and adherence to medications in patient with diabetes [30, 31].

Hence, implications for the Thai context should train health workers and multidisciplinary teams with an explicit focus on eliciting patient’s feelings, beliefs and concerns about illness, and involve patients and their families in decision-making [32]. Rezaei et al. suggest that gaining the patient’s trust can promote the relationship and medical teams should attempt to tailor care programs to each patient based on their lived experiences and unique challenges to increase their adherence to treatment [33]. An easy plan of treatment, specific to each patient’s lifestyle, and acknowledging an individual’s limitation due to social isolation, age or limited health literacy [28] can help ensure that taking medication is a part of routine, daily activities. In Thailand, there is evidence that the benefits of using a multidisciplinary team to provide holistic care for patient with diabetes may also extend beyond medical adherence [34].

## Limitations

A key limitation is generalizability of findings given a specific study population in northern Thailand. However, we found that these factors are similar for other parts of Thailand, and are reassured by similar findings from other populations specifically in Asia and more broadly in LMIC settings. Given that a study nurse conducted the interviews, social desirability bias may have occurred. However, data saturation was a key indicator, and interview data from low and high adherers alike met this threshold. This study may have further benefitted from interviews with physicians and other members of the care team. A more robust measure of adherence and treatment outcomes, such as HbA1c, could have made for a stronger conclusion about a given patient’s true adherence status, however, additional tests would have incurred substantial costs and was not possible for this study.

## Conclusions

Medication adherence in Thai patients with diabetes requires support from both the health care providers and the family. The patient’s perception of the doctor’s concern creates greater patient trust in the health care team. This trust, along with family support, helps deepen patients’ understanding of their disease, accept the chronic nature of the disease, and engenders a positive attitude towards taking medication that can improve medication adherence. Our findings add to literature attempting to understand patient adherence for diabetes, which may inform efforts to address the growing need to improve long-term, chronic disease care in LMIC settings.

## Supplementary information

**Additional file 1.**

## Data Availability

Data generated or analyzed during this study are included in this published article and its supplementary information files.

## Abbreviations

ADLs: Activities of daily living
HbA1C: Glycated hemoglobin or hemoglobin A1C
HCT: Health care team
LMIC: Low-and middle- income countries
T2DM: Type 2 diabetes mellitus
WHO: World Health Organization
