# Supplementary material for: Exploring perceptions, attitudes and beliefs of Thai patients with type 2 diabetes mellitus as they relate to medication adherence at an out-patient primary care clinic in Chiang Mai, Thailand
Source: BMC Fam Pract. 2020 Aug 21;21:173. doi: 10.1186/s12875-020-01233-7 (PMC7442984; doi:10.1186/s12875-020-01233-7)
Supplement: Supplementary file 1 — Additional file 1. [file 12875_2020_1233_MOESM1_ESM.docx]

| **Themes** | **Quote** | **Case** |
| --- | --- | --- |
| 1. Attitude to disease | “I felt fatigue and my head was dizzy and felt tense all day. I had tried herbs for 2 days but they didn't work well. I didn’t know what was happening to me. I felt really sick. I never had these symptoms before, so I went to the doctor. At that time, I thought that I needed to be admitted to the hospital. After the doctor checked and found that my blood sugar was 400 [mg/dl] and my blood pressure was around 200 [mm Hg]...I have continued the medication regularly. I am afraid to forget taking it because I don't want my blood sugar to increase again. Since being diagnosed with diabetes, I have good control of my diet. I don't eat snacks, sweets. I changed from sticky rice to normal rice. I have increased vegetables and decreased fried food**.”** | **#6**: **68 year-old female,**  **high adherence** |
|  | "I was diagnosed with diabetes about 7 years ago after a regular checkup. I was not surprised as I was quite fat at that time. I wasn’t very concerned because I thought my health was very good and I never had any abnormal symptoms. So I decided not to take any medication or see the doctors for 2 years. Then it was suddenly worse when I suffered a stroke. At that time, I thought it was a really terrible condition. I could not live without help from other people, even get dressed or button my shirt. That caused me great concern…I think diabetes is just as bad as cancer. Taking medication can help me live longer, however, I’m concerned that medication can impair my kidneys. Anyway, the doctor said if I don’t take medication, my kidney could be destroyed anyways.” | #**8: 41 year-old male,**  **high adherence.** |
|  | “I didn't have any abnormal symptoms. I came for regular checkup and found that my blood sugar was high.... something like that. I wasn’t very concerned much about being DM because it didn't have any symptom and it just control blood sugar. My aunt also has DM. I believe that it is genetics disease because she was diagnosed even she didn’t drink alcohol or eat sweet diets. Another aunt of mine died from very high blood sugar which was around 700...Right now, I still don't have any abnormal symptom. I am ok with taking medication I thought it may help when can't control diet..... I usually forgot taking pills at evening when I drink alcohol and sometimes from traveling to another province. Anyway, I still take pill on the next day or when I come back home. I don't think much about missing these pills …it was just a few tablets**.”** | **#4: 67 year-old male,**  **low adherence** |
| 1. Attitude to treatment | “I take antidiabetics medication 4 tablets and also one for hypertension and another one for hyperlipidemia for a total of 6 tablets a day. I usually leave antidiabetics medication for the evening. I don't want to take it. I think it is too much. If I didn't have other diseases, I won't have any problems with taking it. I also take herbs and hope that if my blood sugar get better I can stop taking these antidiabetic drugs. I fear that my kidneys will be destroyed. I saw people get hemodialysis from renal failure. I fear to be like that. The doctor told me to take medication regularly, but if I forget just sometime at that evening--it will not be a big problem, right?” | **#22 : 57 years old female,**  **low adherence** |
|  | “I know I have to take antidiabetic medication forever. This disease cannot be cured. I will take it regularly until I die. I meet the doctor at every appointment and take the pills when I return home. If I have to visit my daughter in Japan I will ask for more pills. I never forget to take it. I take 6 pills for diabetes and 1 for dyslipidemia.”  Facilitator: “Do you think is it too many?”  “The amount of medication is lower than before....It's necessary to take them. Some people who don't have other diseases might take fewer pills than me. It's reasonable because I have underlying disease. I want to live a long with my family so I can take care of them.” | **#1: 76 years-old female,**  **high adherence** |
|  | “Right now, I take 2 tablets for diabetes, 1/2 tablet for dyslipidemia and 1/2 tablet for hypertension. I also take several herbs. My family suggests that these herbs could lower blood sugar. My sister was diagnosed with diabetes and used to take anti-diabetic medication and suffered many side effects. After taking herbs, these abnormalities had gone and their blood sugar was controlled. The first time I took medication, I didn’t feel good, sometimes I had palpitations and dizziness. It was too many pills, my body couldn't tolerate it, so I stopped taking them for a while. After that, the doctor adjusted the medication and I had to be admitted to the hospital several times from side effects. These days, I am fine. I am OK with the amount of my medication. I take less than before. In fact, I don't want to take anti-diabetic medications. I think it can accumulate in my body and end up with kidney disease or other consequences. I saw my uncle, he took a lot of medication around 6-7 tablets every day until he suffered renal failure and required a kidney transplant. I notice that anyone who takes medication, they are never cured from diabetes even taking it every day...like my uncle. You know...he had taken it every day. I never saw his blood sugar controlled and worse than that--he had to inject insulin every day**.** | **#15: 49 year-old female,**  **low adherence** |
| 1. Attitude to family support | “When one of us [participant or the participant’s husband] has free time, we will prepare our own medication and each others, so we can know who forgets taking their pills. We usually take medication together after breakfast. He helps me a lot.” | **#9: 60 year-old female, high adherence** |
|  | “My children usually remind me to not eat sweets or fatty foods and to eat more fruits and vegetables. They helped me prepare medication in the beginning but now I can do by myself. They usually remind me not to forget to take medication every day.” | **#1: 76 year-old female, high adherence** |
|  | “I prepare medication by myself, no one helps me. My wife reminds me sometimes....once in a while...It would be nice if she asked me every day. I understand she is busy....has a lot of work to do.” | **#18: 64 year-old male, low adherence.** |
| 1. Attitude to health care team | “I decided to start anti-diabetic medication upon my doctor’s advice. In fact, I can still do only lifestyle modification. I know that I am quite old right now and should do as the doctors suggest mostly. They are specialized in this way, however, I wonder about my own situation of having DM, and ask, “Why do I have this disease?” and, “Is the suggestion from the doctor clear**?”** | **#20: 64 year-old male, high adherence** |
|  | “I don't stress about taking anti-diabetic medication. The number that I should take is up to the doctor’s suggestion. It' s important to believe the doctor, so I take medication regularly. Someone may stop medication by themselves if their blood sugar is improve but not me. I want to hear from my doctor first. They are very nice to me. With these good advice and their support, I feel 100% that I can control my DM. Sometimes I work out-of-town as a tour guide, but I still make sure to take the medication that the doctor prescribed.” | **#23: 59 year-old male, high adherence** |
